# Animal models of brain metastasis

**DOI:** 10.1093/noajnl/vdab115

**Published:** 2021-11-27

**Authors:** Lauritz Miarka, Manuel Valiente

**Affiliations:** Brain Metastasis Group, Molecular Oncology Programme, Spanish National Cancer Research Centre (CNIO), Madrid, Spain

**Keywords:** brain metastasis, experimental models, therapy, treatment toxicity

## Abstract

Modeling of metastatic disease in animal models is a critical resource to study the complexity of this multi-step process in a relevant system. Available models of metastatic disease to the brain are still far from ideal but they allow to address specific aspects of the biology or mimic clinically relevant scenarios. We not only review experimental models and their potential improvements but also discuss specific answers that could be obtained from them on unsolved aspects of clinical management.

## Getting to Know the Enemy: The Need for Good Brain Metastasis Models

The complexity of the metastatic process has long been recognized and is comprised out of different dynamic steps: (a) Initially, carcinoma cells become invasive and leave the primary tumor. (b) Subsequently, they intravasate into the vasculature, where they have to evade immune attack and survive in the circulation. (c) A subset of circulating tumor cells will eventually extravasate into the foreign tissue. (d) Here, they are confronted with a potentially hostile microenvironment, forcing them to adapt and form a hospitable microenvironment, which may happen during a period of latency.^1^ Overall, this is a very inefficient process where the majority of cancer cells leaving the primary tumor will fail along the different steps. Even if considering the very last step of the cascade alone, organ colonization, the numbers of successful metastatic cells could get to 1 in 100 of the cells that completed extravasation in the brain.^2^ The brain microenvironment represents a unique niche: Metastatic cancer cells will encounter tissue-resident cell types such as microglia, oligodendrocytes, astrocytes, and neurons, and will have to cope with distinct metabolic and extracellular matrix characteristics.^3^ Additionally, the brain is shielded by the blood–brain barrier, providing a selective barrier for many molecules coming from the systemic circulation, including therapeutic agents.^3^ Even though the barrier gets transformed into a different entity termed blood–tumor barrier, it still keeps a selective permeability.^4^ Since cancer cells from diverse primary origins home to the brain, the complexity of the microenvironment is complemented by genetic and phenotypic heterogeneity of the brain metastatic cancer cells themselves.^5^ Thus, to decipher the formation of CNS metastasis and discover novel therapeutic options, it is paramount to use preclinical models that faithfully recapitulate the complexity of this multifaceted process. However, no model can cover the wide range of unresolved questions. Choosing the right model therefore depends on the scientific question asked by the investigator: Reductionist models can provide faster and more direct insights, while risking limited relevance in more complex settings, and are therefore more suited for gaining mechanistic insights into specific aspects of tumor biology. On the other hand, models recapitulating the heterogeneity and complexity of human disease and the clinical situation are needed for testing new therapeutics or biomarker approaches.

In this review, we introduce basic concepts of different experimental brain metastasis models and explore which questions they might help answer. While most work in the field has focused on murine models, other model organisms clearly have value in complementing this experimental tool and are therefore shortly summarized.

## Generation of Brain Metastatic Cell Lines

The use of brain metastatic cell lines represents the most widely used approach. Here, cancer cells are routinely isolated from patient material and further propagated in vitro. These cells, termed parental (P), are usually transfected with reporters such as luciferase and a fluorescent protein (eg, GFP), compatible with evaluation of metastatic burden in vivo and by histology, respectively. Since inoculation of these parental cell lines into mice usually does not yield a high number of brain metastasis, brain-seeking clones can be enriched by repeated rounds of in vivo selection. For this, parental cells typically are injected into the arterial circulation (either into the left cardiac ventricle—intracardiac—or into the carotid artery) and few cells will follow the blood stream and colonize the brain. Subsequently, these cancer cells will be recovered from the animal’s brain, expanded in vitro and re-injected into mice. Repeating this selection process for 3–5 times increases the cancer cells ability to form lesions in the brain and eventually a brain-tropic cell line (BrM) can be established, that upon injection reliably generates brain metastasis.^6^ Of note, while more aggressively metastasizing derivatives are generated by dissecting bioluminescence-positive brains, dissection of bioluminescence-negative brains has been used to establish also indolent BrM cell lines.^6^ Utilizing this in vivo selection approach, a wide range of both human and murine BrM cell lines could be obtained, representing the most frequent primary origins and oncogenomic profiles.^7^ Most of these cell lines and their respective characteristics are summarized in the BrMPanel, a resource provided by a consortium of brain metastasis researchers.^7^

In order to generate brain metastasis in vivo, these cells are typically inoculated into animals in 2 different ways:

### Systemic Inoculation

For this injection route, BrM cells are injected either into the left cardiac ventricle or, less common, into the carotid artery. Following intracardiac inoculation, BrM cells are distributed with the arterial blood flow into the brain, but also generate significant extracranial metastatic burden (eg, in the lungs, bone, liver). This can be particularly problematic in syngeneic hosts, in which intracardiacally injected mice usually reach their humane endpoint due to extensive visceral metastasis, before brain metastatic lesions reach a critical size. Extracranial metastasis can be avoided by inoculating cancer cells into the carotid artery. However, intra-carotid inoculation is a much more invasive procedure, which requires surgery and a bigger time investment, therefore may not be as readily applicable as intra-cardiac injection.^8^ While both ways include the strong selection step of extravasation, earlier steps of the metastatic cascade such as invasion and the possible formation of a premetastatic niche are neglected.^9^

### Intracranial Inoculation

Here, BrM cells are injected directly into the brain, usually using a stereotactic apparatus. This provides the advantage of generating a singular, precisely located established lesion, making it suitable for questions in which the healthy hemisphere can be used as an endogenous control^10^ or for syngeneic models in which a longer period of time (eg, for treatment) after establishment of the lesion is needed.^11^ However, intracranial inoculation does not faithfully recapitulate the metastatic cascade, since cancer cells are not required to extravasate. Although it can be used to study the interaction of cancer cells with the brain microenvironment, attention should be paid to the fact that the injection itself already induces neuroinflammation.

Of note, by all of the listed routes of inoculation, BrM cells are injected into a tumor naïve animal without primary tumor, which does not reflect the clinical scenario. This might influence the response to therapy, in particular immunotherapy.^12^ One possibility to circumvent this challenge is the simultaneous co-implantation of orthotopic and brain metastatic tumors.^12^

### Spontaneous Models of Brain Metastasis and Genetically Engineered Mouse Models

Ideally, an experimental model of brain metastasis would require cancer cells to undergo all steps of the metastatic cascade, either from orthotopically injected tumor cells, such as the mammary fat pad for breast cancer or subdermal for melanoma, or from genetically engineered mouse models, which spontaneously form tumors after genetic manipulation of oncogenes or tumor suppressors. Unfortunately, few cancer cell lines spontaneously form intracranial lesions from orthotopic injection (Table 1). Similar to brain metastasis in patients, their occurrence is rather late in the course of disease, often requiring surgical removal of the primary tumor in order to prevent mice from reaching the humane endpoint prematurely due to extensive extracranial disease.^9^ Additionally, the incidence of brain metastasis in these models is low, causing high experimental variability and therefore a need for bigger cohorts of mice. In spite of these drawbacks, these models are necessary to test therapeutic approaches aiming at the prevention of metastasis, the natural selection process of metastatic clones from the primary tumor, as well as the influence of the latter on the metastatic niche. While these models are scarce, for the common primary tumor types there are models available.

**Table 1.** Spontaneous Models of Brain Metastasis

Model	Cancer Type	Specie	Host	Primary Tumor/ Orthotopic Inoculation	Surgery of Primary Needed	Time to Brain Metastasis	Detection of Brain Metastasis	Incidence of Brain Metastasis	Reference
MDA-MB-453	Breast cancer (Triple negative)	Human	Rag2^-/-^; Il2rg^-/-^	Subcutaneous and orthotopic inoculation (Fat pad)	Not described	Not described	Histology	Not described	Nanni et al.
BT-474	Breast cancer	Human	Rag2^-/-^; Il2rg^-/-^	Subcutaneous and orthotopic inoculation (Fat pad)	Not described	Not described	Histology	Not described	Nanni et al.
MDA-231	Breast cancer (Triple negative)	Human	NSG	Orthotopic inoculation	No	2 months	Histology	100%	Puchalapalli et al.
CN34BrM	Breast cancer (Triple negative)	Human	NSG	Orthotopic inoculation	No	2 months	Histology	90%	Puchalapalli et al.
SUM1315	Breast cancer (Triple negative)	Human	NSG	Orthotopic inoculation	No	3 months	Histology	11%	Puchalapalli et al.
TBCP-1	Breast cancer (Her2+)	Mouse	BALB/C	Orthotopic inoculation	Yes	7 weeks	Histology	60%	Nagpal et al.
Dct::TVA;BrafV600E; Cdkn2alox/lox; Ptenlox/ lox + RCAS-Cre and RCAS-myrAKT1	Melanoma	Mouse	Mixed C57Bl/6 and FVB/N	GEMM (spontaneous primary tumor)	No	6 weeks	Histology	79%	Cho et al.
RMS; sBT-RMS	Melanoma	Mouse	C57Bl/6	Orthotopic inoculation	Yes	RMS (3-6 months); RMS-BrM (1.5 months)	Histology and qRT-PCR	23% (RMS); 64% (sBT- RMS)	Schwartz et al., Doron et al.
Rbnull/Trp53null	Small cell lung cancer	Mouse	Not reported	GEMM (spontaneous primary tumor)	No	7 months	Histology	Not described	Meuwissen et al.

### Breast Cancer

Few breast cancer cell lines (MDA-MB-453, BT-474, MDA-231, CN34BrM, SUM1315) have been reported to spontaneously form brain metastasis after orthotopic injection, but not in commonly used host nude mice; Instead the use of more permissive hosts lacking NK-cells, such as NSG or Rag2^-/-^; Il2rg^-/-^ mice, was necessary.^13,14^ In a immunocompetent host, Nagpal et al. recently reported a HER2+ breast cancer cell line derived from spontaneous BALB/C mammary tumors, which after orthotopic injection and subsequent resection of the primary tumor avidly metastasizes to the brain (60% of mice).^15^

### Melanoma

Activation of *Akt1* by either addition of a N-terminal myristoylation sequence^16^ or expression of constitutively active *Akt1*^E17K17^ in an autochthonous melanoma mouse model in the context mutated *Braf*^V600E^ and loss of *Pten* and *Cdkn2a* leads to brain and extracranial metastasis in an otherwise nonmetastatic model. A second model has been described for the melanoma cell line RMS, derived from spontaneously arising skin tumors in *Ret* transgenic mice,^18^ which after subdermal injection yields brain metastasis 3–6 months after surgical removal of the primary tumor in 23% of injected mice.^9^ Isolation of and subsequent culture of cells from these spontaneous melanoma brain metastasis gave rise to a brain-trophic cell derivate of the RMS cell line, developing brain metastasis in 64% of mice upon subdermal re-injection.^19^

### Small-Cell Lung Cancer

Inactivation of *Rb1* and *Trp53* are frequently found mutations in small-cell lung cancer and concomitant loss of *Rb1* and *Tp53* in mouse lungs leads to a high incidence of SCLC in these mice, recapitulating the aggressive phenotype observed in humans including the formation of extracranial and brain metastasis.^20^

## Patient-derived Xenograft (PDX) Models

While the use of established cell lines has been the main methodical approach is brain metastasis research, it becomes increasingly recognized that these cell lines do not recapitulate the broad spectrum of genomic alterations in human cancers, particularly when it comes to the genomic evolution under different kind of therapies that primary tumor undergo before or simultaneously metastasizing to the brain or, in the case of lung cancer, the extensive tobacco-induced mutagenetic landscape.^21,22^ Furthermore, established cell lines often suffer from genomic drift during passage and acquire additional mutations in vitro over time.^23^

For PDX models, patient-derived tissue could be implanted directly into mice or patient-derived cells might be propagated in vitro for a very limited number of passages (usually < 10), during which they can be engineered with different reporters (Luciferase, GFP) before being injected into immunodeficient mice (NSG). Compatibility with *in vitro* passage is not granted and efficacy has been estimated between 24% and 48.8% depending on the amount of viable cancer cells in the surgical sample, ability of the cells to form colonies in the first culture, and the degree of senescence affecting cancer cells.^24^ Most brain metastatic PDX models are derived from either systemic^25^ or intracranial^26^ inoculation, but few models have been reported to spontaneously metastasize from orthotopic (subdermal for melanoma^21^ or mammary fat-pad for breast cancer^27^) injection. Of note, a brain-metastatic SCLC PDX model has recently been established from subcutaneous injection.^22^ This is of considerable importance, since human SCLC patients have an extremely high propensity to develop brain metastasis, but so far there are no human cell lines (and only one insufficient mouse model described above) that recapitulate this important feature of SCLC.

Remarkably, PDX-based brain metastasis accurately represent the histological, genomic and molecular features of their parental tumor,^26^ providing the opportunity to utilize these models as patient avatars to develop and validate more personalized therapies. The advantage of PDX models for testing of novel targeted therapies for rare, but potentially actionable genomic vulnerabilities has recently been illustrated for both *ROS1* and *MET* mutated NSCLC.^28,29^ Both of these mutations represent distinct molecular subtypes of NSCLC that frequently metastasize to the brain,^30,31^ but have not been modeled yet by conventional cell lines. Treatment of experimental brain metastasis generated from orthotopically inoculated PDX from patients harboring these mutations with the targeted therapies repotrectinib^29^ or savolitinib,^28^ respectively, resulted in intracranial tumor growth inhibition and in the case of *ROS1* mutated brain metastasis treated with repotrectinib even in doubled survival time of mice.

In contrast to established cancer cell lines, so far, the utility of PDX models for functional testing has been limited because of the inability to perform genetic targeting in these tumors. However, recent development of novel Crispr-Cas9 editing methods, that do not require in vitro culture for selection of transduced cells, enables targeted genome editing in PDX,^32^ augmenting their power.

Another approach to harness the utility of PDX in brain metastasis research is the derivation of PDX models from circulating tumor cells of brain metastasis patients (also CDX). These precursors of metastasis isolated from the blood of the patient have been shown to recapitulate their organ tropism to the brain when injected intracardiacally in mice,^33^ offering the unique opportunity to test preventative therapeutic approaches. This might be particularly interesting since a subset of brain metastasis patients is not eligible to undergo neurosurgery and therefore no surgical specimen for PDX derivation is available in these patients.

One limitation of PDX models is the necessary use of immunocompromised hosts, considering the growing interest in the use of various kinds of immunotherapies in brain metastasis.^5^ Humanized mouse models comprised a severely immune-suppressed host (eg, NSG or NOG mice) in which a functional human immune system is reconstituted by engraftment of either human peripheral blood mononuclear cells or human hematopoietic stem cells.^34^ While these models are constantly advancing and have been able to generate some insides into immunotherapy responses in other tumor entities,^35^ their generation remains labor-intensive and costly. Therefore, these models have yet to be introduced into brain metastasis research.

## Other Animal Models of Brain Metastasis

**Figure 1.**
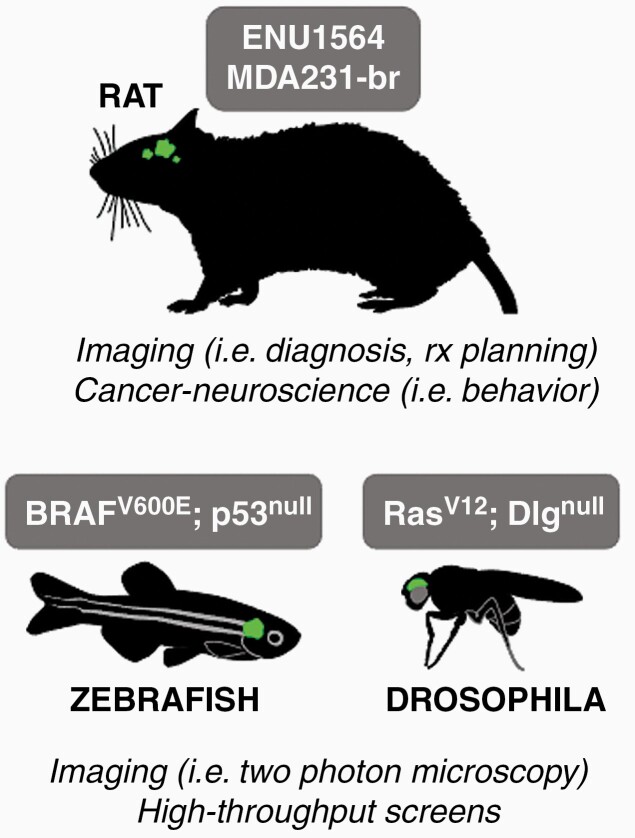
Alternative brain metastasis models. Different models of brain metastasis reported in the literature are described including the cell line used (Rat, zebrafish) or the genetic modification leading to brain metastasis. Each of these organisms provide specific advantages for analyzing brain metastasis.

While mouse models have been the most widely studied in the field of brain metastasis, other in vivo models may complement insights gained from mouse models or may even be better suited for certain research questions (Figure 1).

There has been at least 2 established brain-metastatic rat models: The syngeneic breast cancer cell line ENU1564, derived from a *N*-ethyl-*N*-nitrosourea–induced mammary adenocarcinoma in female Berlin-Druckrey IX (BD-IX) rats,^10,36^ which can be injected systemically or intracranially, as well as the human breast cancer cell line MDA-321br, which can be used in nude rats.^37^ These models have proven to be particularly superior for the study of imaging techniques, since the rat brain is larger than a mouse brain and therefore offers a better spatial resolution.^36,37^ Advanced imaging plays an essential role in the clinical management of brain metastasis patients, not only for establishing the diagnosis, but also for treatment planning of radio-therapy and accurate determination of treatment response. Particularly the latter purpose provides a considerable challenge for clinicians, as early recognition of treatment failure can be decisive to initiate salvage therapies before clinical onset of symptoms associated with tumor re-growth, but often is difficult to differentiate on conventional imaging platforms.^38^ Thus, the further improvement of brain metastasis rat models could serve as a platform for preclinical development of novel imaging techniques.

Another area in which rat brain metastasis models may prove as useful is the emerging field of cancer neuroscience^39^: There is a growing appreciation that brain tumors, including brain metastasis, are able to integrate into neuronal circuits of the CNS.^40–42^ While most studies so far have reported the influence of neuronal activity on cancer cell proliferation, the clinical observation of brain tumor patients experiencing different degrees and types of neurological symptoms, independent of location or size of their respective tumor, indicates that this cancer-neuronal crosstalk may be bi-directional and induces neuro-cognitive impairment.^43^ In order to dissect the impact of brain metastasis on cognition and behavior, specific task-related cognitive tests such as the morris water maze (assessing spatial learning and memory^44^) or attentional set-shifting task (assessing executive functioning^45^) need to be performed. Mice are much easier stressed by human contact than rats and this, as well as other “non-cognitive” distractors often cofound their performance in behavioral tests, while rats perform more stably over time.^46^ In conclusion, research into the cognitive impairment in brain metastasis patients could benefit from using rats as an additional model organism.

Of note, 2 nonrodent models have been used for brain metastasis research: The zebrafish (*Danio rerio*) has emerged as a powerful tool to study the metastatic cascade in vivo, owing to its optical transparency that in combination with fluorescently labeled cancer cells allows quantitative assessment of the spatial-temporal dynamics of metastasis on a single cell level.^47^ Since zebrafish are easy to breed and to genetically manipulate, they represent an ideal model organism to perform high-throughput genetic screening for possible mediators of metastasis.^47^ Using a spontaneous zebrafish melanoma model, generated by expression of mutant *BRAF*^V600E^ under a melanocyte promoter in *p53*-deficient zebrafish,^48^ a transplantable metastatic cell line was established, enabling the functional manipulation of both cancer-cell intrinsic and microenvironmental determinants of the metastatic process.^47^ Also, Stoletov et al. used the zebrafish model in combination with the mouse breast cancer cell line 4T-1 to uncover the role of connexins during the early metastatic colonization of the brain.^49^

Another nonrodent model organism that was adapted for brain metastasis research is *Drosophila melanogaster*.^50^ Here, researchers made use of a fly model carrying overexpressed oncogenic *Ras*^V12^, inactivated of the cell polarity gene *Dlg* and GFP in the imaginal eye disc, leading to tumor development in the Drosophila eye disc and consequent invasion into adjacent brain tissue.^50,51^ Similar to zebrafish, Drosophila offers an ideal platform for high-throughput genetic screening by simply crossing the above-described fly line with any RNAi fly line. Utilizing this approach to interrogate a 108-gene signature obtained from RNA-sequencing of small versus big mouse brain metastasis, Howe et al. identified Rab11b as a mediator of metastatic adaption in the brain,^50^ necessary for protein recycling of Integrin-ß1, which in turn enables the successful interaction of cancer cells with the brain microenvironment.

Overall, nonrodent model organisms offer attractive advantages compared with rodents, such as low maintenance effort and cost, simple ways to generate transgenics and, in the case of zebrafish, the unique ability for whole-body in vivo imaging. While most of the human protein-coding genome is conserved in these model organisms, rendering them ideal for genetic screening, it should be noted that they exhibit significant physiological and anatomical differences compared to human. Depending on the research question, these models could complement the research performed in rodent models.

## Including Local Therapies Into Brain Metastasis Models

**Figure 2.**
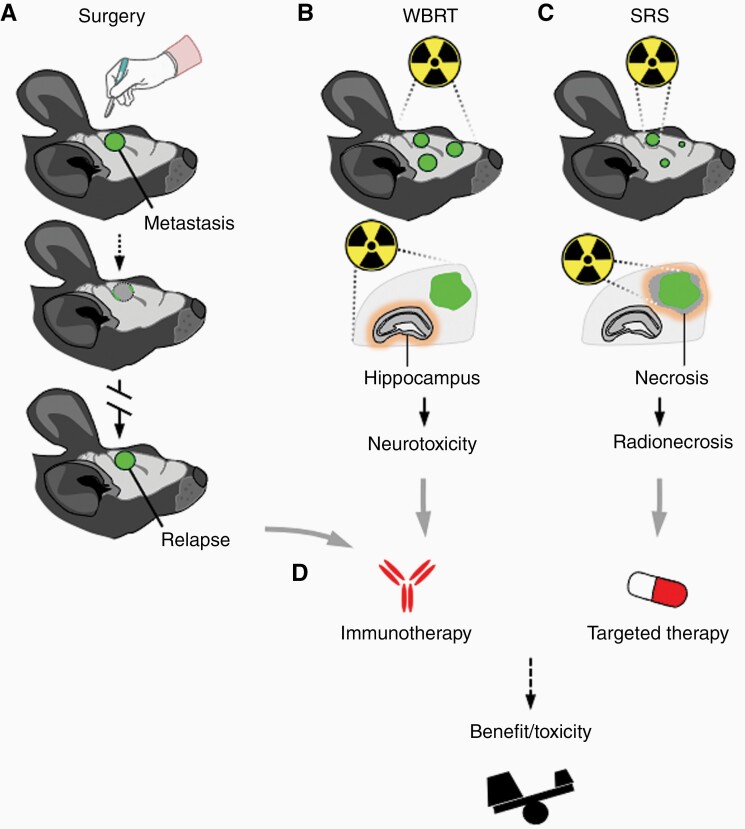
Experimental models incorporating local therapies. Both neurosurgery and radiotherapy have been applied to experimental brain metastasis models in vivo. These approaches could help to understand the biology of local relapse (a), neurotoxicity related to whole brain radiotherapy (WBRT) (b), and the radionecrosis that could be associated with stereotactic radiosurgery (SRS) (c). In addition, these models could be used to evaluate the benefit versus increased toxicity of combining local and systemic therapies (d).

Neurosurgical resection is a cornerstone of the clinical management of brain metastasis patients, often providing immediate relief for neurological symptoms as well as a benefit in survival, at least for a subgroup of patients.^38^ However, local or distant intracranial recurrences are frequent and represent a considerable challenge.^52^ While neurosurgery and subsequent relapse has been established in mouse models of pediatric brain tumors^53^ and recently of glioblastoma,^54^ similar approaches mimicking this clinically relevant scenario in brain metastasis are lacking. To this end, our group recently has developed a novel in vivo model of local relapse after neurosurgical resection of a single brain metastasis (Figure 2), providing an experimental platform to test therapeutic approaches in a truly adjuvant setting as well as the experimental opportunity to study the biology of relapse.^55^ Given the recent clinical interest in immunotherapy in brain metastasis, it would be desirable to extent this approach to syngeneic models, in order to dissect the impact of neurosurgery and subsequent treatment with steroids on the immune-infiltrate of the brain.

In brain metastasis patients, neurosurgery is usually followed, or in some patients even completely replaced, by different radiotherapy modalities.^38^ Historically, whole-brain-radiotherapy (WBRT) used to be the gold standard in the management of brain metastasis, owing to its ability to control both local and distant intracranial disease. Recent clinical trials however have questioned this approach, given that compared to best supportive care no benefit in survival could be detected, at the cost of high neurotoxicity.^56^ Given this apparent resistance to irradiation, experimental models of brain metastasis also ought to incorporate this therapeutic modality, ideally in combination with neurosurgery, to faithfully recapitulate the clinical scenario. A small number of studies described the use of WBRT: Two studies have used a single dose of irradiation in breast and lung cancer brain metastasis models.^57,58^ While the first demonstrated an anti-tumoral effect when irradiation was applied to micrometastasis, both confirmed the clinically observed radio-resistance of established metastasis. In patients, WBRT is usually not given as a single-dose, but delivered using hypofractionated protocols.^38^ To this end, Smart et al. applied WBRT in 10 fractions of 3Gy to breast cancer brain metastasis in vivo, a schema closely mimicking the one given in the clinic.^59^ Here, fractionated irradiation failed to affect tumor growth of established brain metastasis.

Owing to the ambiguity of WBRT, clinical practice rapidly adapted stereotactic radiosurgery (SRS), a radio-therapeutic approach where a high dose of irradiation is delivered precisely to the metastatic lesion, while sparing healthy surrounding tissue, therefore avoiding neurocognitive toxicity.^38^ This mode of irradiation delivery has also been integrated into preclinical models of brain metastasis, using cone-beam computed tomography and magnetic resonance imaging for treatment planning and single arc radiation exposure to deliver doses raging from 18 to 40Gy.^60^ In this context, it was shown that tumor breakdown by SRS, rather than imprecise radiation, leads to cognitive decline after irradiation.^60^ Interestingly, the same authors were capable of achieving long term survival and eradication of brain tumors using a rat gliosarcoma model, enabling the study of late effects of irradiation on cognition in the context of a brain tumor.^60^

Overall, brain metastasis usually occurs late in the evolution of disease and therefore patients have undergone multiple lines of treatment for their primary tumors and subsequently will receive either neurosurgery, radiation, systemic treatment or a combination of these approaches for treatment of their brain metastasis. This complexity is not reflected in animal models of brain metastasis, which mostly are treatment naive. It is therefore imperative to include these modalities in preclinical models not only to mimic the clinical situation more closely, but also to study the biology of relapse after therapies such as neurosurgery or irradiation. Furthermore, animal models can serve as a preclinical discovery platform to dissect the impact of different treatment modalities on each other, such as radiotherapy and immunotherapy, which has recently been addressed in experimental models of brain metastasis.^61^

## Systemic Therapies

Traditionally, systemic cytotoxic drugs only play a limited role in the clinical management of brain metastasis patients, due to their failure to penetrate the blood–brain barrier (BBB). Even though it has been demonstrated in experimental breast to brain metastasis models that the BBB is compromised in most metastatic lesions and these therefore enrich cytotoxic drugs more than an unaffected brain, these concentrations were not sufficient to show an effect on intracranial tumor growth. ^62^ Despite these discouraging results, few studies could show benefits from chemotherapeutic agents have shown promising results in preclinical models: When temozolomide, a BBB-permeable alkylating-agent frequently used in primary brain tumors, was given to mice 3 days after intracardiac inoculation of breast cancer cells, it completely prevented formation of brain metastatic lesions and enabled long-term survival of treated mice.^63^ This benefit however was not observed when Temozolomide was given to mice when metastases were already established or when O^6^-methylguanin-DNA-methyltransferase (MGMT), a DNA-repair enzyme known to confer resistance to alkylating-agent induced damage, was overexpressed. While in clinical trials, Temozolomide so far was not effective in brain metastasis patients, evaluation in a preventive setting is under ongoing investigation.^64^

Next to chemotherapy, considerable effort has been invested to develop targeted therapies which inhibit specific molecular drivers of cancer progression, providing distinct subsets of patients carrying these mutations with a significant benefit of survival.^38^ This has particularly important implications for the treatment of brain metastasis, since patients carrying some of these mutations have a higher propensity to develop brain metastasis (such as *EGFR*-mutant NSCLC or *HER2*+ breast cancer). While some of these agents have shown promising intracranial activity, many phase III randomized trials on the efficacy of targeted therapies still specifically exclude brain metastasis patients.^65^ Thus, the use of animal models provides an opportunity to test novel targeted therapies for their intracranial activity and if positive, promote the inclusion of brain metastasis patients in trials evaluating these agents. A wide range of targeted therapies has been tested in experimental models of brain metastasis; since this has recently been extensively reviewed elsewhere,^66^ here we will only give examples for the most common subtypes of brain metastatic cancers.

When experimental brain metastasis were established from intra-carotid injected human NSCLC cells harboring a *EGFR* exon 19 deletion, the third-generation EGFR inhibitor Osimertinib achieved sustained tumor regression and extended mice survival.^67^ This benefit of Osimertinib was later recapitulated in a randomized phase III trial, in which the compound showed good intracranial activity and was able to prolong disease-free survival in patients with CNS disease.^68^

HER2+ breast cancers can be targeted by a variety of different agents, with most of them showing a varying degree of intracranial activity.^38^ While monoclonal antibodies such as trastuzumab have low permeability across the BBB and therefore play a limited role in the management of brain metastasis, the more recently developed pan-HER kinase inhibitors tucatinib and neratinib have shown more promising intracranial response rates, particularly in combination with capecitabine.^69^ To this end, Nagpal et al. showed in a spontaneously metastasizing HER2+ breast cancer model that neoadjuvant neratinib monotherapy significantly reduces the incidence of brain metastasis, suggesting that this may be a more suitable clinical setting than late intervention.^15^

In melanoma, approximately half of advanced-stage patients display mutated *BRAF*, leading to hyperactivated BRAF kinase and enhanced MAPK signaling.^70^ Several MAPK inhibitors for targeting *BRAF*^V600E^-mutated melanomas, such as dabrafenib (targeting BRAF) or trametinib (targeting MEK), have been developed and already showed intracranial response rates as monotherapies, which was further improved by combining both dabrafenib and trametinib together.^71^ Despite the high frequency and therefore clinical importance of *BRAF*-mutated melanoma brain metastasis, preclinical models testing this treatment modalities are scarce. In a PDX brain metastasis model established from intracranially injected *BRAF*^L597S^-mutant melanoma cells, trametinib monotherapy was able to slow tumor growth, but not to extend survival. Combination of both trametinib and dabrafenib though significantly improved survival of mice, when compared with monotherapy or vehicle, confirming the clinically observed superior activity of combinational therapy.^70^

In summary, there is a wealth of preclinical evidence suggesting that testing both chemo- and targeted therapies in animal models of brain metastasis is able to recapitulate most of the clinical responses to these therapies and therefore can serve as a platform to test new agents as well as to design combinatorial treatments. Here, it is imperative to use approaches that model the most likely clinical setting, which is established, heavily pretreated brain metastasis. Looking at both the use of lapatinib in *HER2*+ breast to brain metastasis and vemurafenib in *BRAF*-mutant melanoma brain metastasis, trials have shown these agents to be less effective in previously irradiated patients.^38^ So far, this has not been addressed in preclinical studies modeling these therapies in experimental brain metastasis. Furthermore, in the landscape of available brain metastasis models, distinct genomic subsets, which are already treated with specific targeted therapy in the clinic, such as *RET*-mutated lung cancers, are currently still underrepresented or not available. In addition, taking into account the potential existence of genomic divergence between the primary tumor and derived metastases,^72^ including brain metastases,^73,74^ should be incorporated in experimental models to determine the contribution to differential responses to targeted treatments between the primary tumor and systemic disease. However, broadly used metastasis models (ie, organotropic cell lines) do not recapitulate this genomic evolution^75^ and, consequently, special attention should be paid to the analysis of other models that have a slower evolution from the development of spontaneous primary tumor and subsequently derived brain metastasis^9,16,20^ to evaluate whether they are compatible with such approaches.

## Modeling Treatment-related Toxicity

**Figure 3.**
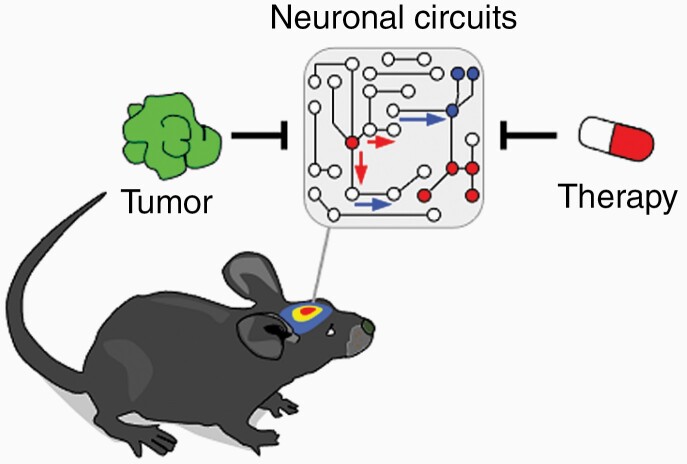
Impact of brain metastasis and their treatment in brain function. Both the tumor but also systemic and local treatments might have an impact in neuronal communication that could generate neurocognitive defects, which are highly prevalent among cancer patients with brain metastasis.

Given that modern therapeutic strategies increasingly enable improved cancer outcomes and long-term survivorship, treatment-related cognitive and behavioral impairments become more evident and contribute to suboptimal quality of life.^76^ While historically cognitive impairment was mostly associated with chemotherapy and whole-brain-radiotherapy, newer therapeutic modalities such as targeted- or immunotherapy have very different effects on cognition and brain function in general, and therefore represent new challenges in the management of neurotoxic side effects. Studying the interaction of therapeutic modalities and the CNS in preclinical models will enhance the understanding of molecular underpinnings of their neurotoxicity and facilitate the development of strategies to mitigate these symptoms (Figure 3).

Whole-brain-radiotherapy is often followed by late-onset adverse effects like memory loss, confusion and leukencephalopathy.^38^ When tumor-naive mice were treated with 10Gy cranial irradiation, impairments of mice behavior and execution of cognitive tasks related hippocampal learning and memory were observed, which was accompanied by microglia activation.^77^ Cognitive deficits could be ameliorated by administration of either a CSF1R-inhibitor, depleting microglia, or microglia-specific deletion of C1q, together suggesting that radiotherapy-induced activated microglia contribute to cognitive dysfunction by complement-mediated synaptic loss.^77,78^ Other studies in rats have shown that treatment with 10Gy irradiation specifically inhibits the proliferation of hippocampal precursor cells by disrupting their neurogenic niche, ablating adult neurogenesis, which is thought to be important for normal hippocampal function.^79^ To this end, advances in radiation technology enable clinicians now to physically limit the radiation dose delivered to the hippocampus during whole-brain-radiotherapy, a technique called hippocampal sparing irradiation (HSI). When HSI was applied to mice and compared to regular WBRT, HSI-treated animals performed significantly better in behavioral tasks, consistent with recent clinical trials showing that HIS mitigates cognitive decline in brain metastasis patients receiving cranial irradiation.^80^

While stereotactic radiosurgery compared to WBRT does not cause a notable increase in neurocognitive toxicity, it can cause radionecrosis in 13%–14% of patients.^81^ Few murine models of radiation necrosis have been reported, although most of them with the purpose of developing novel imaging techniques to distinguish between tumor recurrence and radiation necrosis.^82^ Here, authors of one study treated the left hemisphere of mice with 50Gy radiation to develop radiation necrosis and subsequently administered bevacizumab, a VEGF antibody frequently used in the clinic to treat radiation necrosis.^83^ While bevacizumab significantly decreased necrotic lesion volume on imaging, histologically typical necrosis pathology was still present up to 12 weeks after irradiation.^83^ Of note, this and other studies utilized tumor-naive mice to study the effects of SRS-associated radiation necrosis; this may not fully represent the clinical phenotype, since another study observed that treating actual experimental brain tumors with SRS results in persistent cognitive deficits due to tumor disintegration and neuroinflammation, while SRS of healthy brain tissue only lead to transient effects.^60^ Clinical experience has shown that the risk of radionecrosis after SRS is increased when combined with immunotherapy.^38^ Unfortunately, this additive effect and its possible implications and adverse effects have not been addressed in the few preclinical studies that evaluated the combination of SRS and immunotherapy.^84^

In general, adverse effects of immunotherapy in brain metastasis specifically have not been considered in preclinical studies, even though the use of immune checkpoint inhibitors because increasingly popular in patients with brain metastasis.^38^ Clinically, about 4% to 6% of patients treated with one immunotherapy agent and up to 12% treated with 2 immunotherapeutic drugs experience neurological impairments, with symptoms ranging from headaches to meningitis.^85^ In experimental models of lung and colorectal cancer, although without brain metastasis, treated with a systemic CTLA-4 inhibitor and peripheral irradiation, changes in behavior and cognitive impairment were observed in tumor-bearing mice receiving therapy, compared with those mice without.^86^ These adverse events were accompanied by activation of microglia and general neuroinflammation, pointing to a similar mechanism as described for cranial irradiation-associated cognitive decline.^86^

## Future Advances in Modeling Brain Metastasis

**Figure 4.**
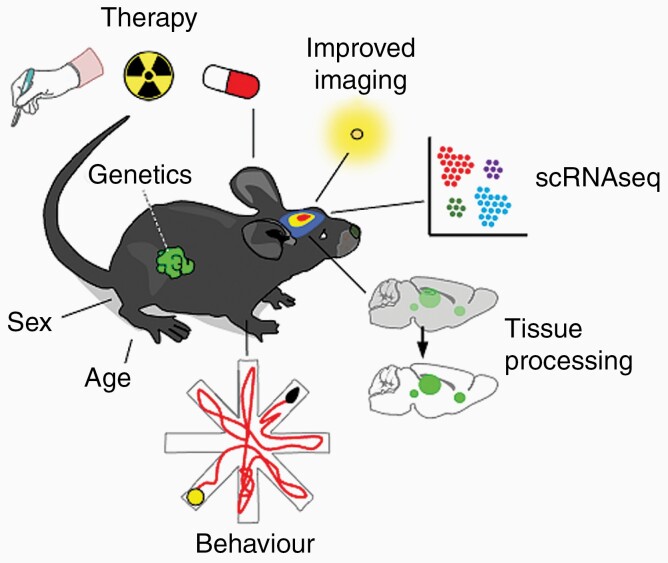
Technical aspects to improve brain metastasis models. Incorporating therapies is highly necessary to recapitulate the disease in patients. In addition, the presence of a primary tumor with known genetics, considering sex and age as important variables, the use of improved molecular imaging resources (ie, next generation of luciferases), single-cell technologies to deconstruct the complexity of the brain metastasis associated microenvironment, advanced tissue processing technologies (ie, tissue clearing) so intact metastasis could be studied, and evaluate the impact of brain metastasis in the whole organisms (ie, behavior) will certainly increase the quality of the evaluation of metastatic disease in the brain.

While our knowledge of the molecular underpinnings of brain metastasis is steadily increasing and this reflects in continuously improved preclinical models, new approaches are needed to more faithfully recapitulate human disease in its heterogeneity and evolution under treatment. Additionally, new technologies are emerging that allow to dissect tumor biology at unprecedented resolution, enabling the discovery of new druggable targets in both cancer and microenvironmental cells (Figure 4).

### Getting Closer to Reality

Systemic or local injection or brain-tropic established cell lines is still the most common method to study brain metastasis in a preclinical setting, however these approaches do not recapitulate the complete metastatic cascade, the heavily pretreated host or the genomic complexity of the human disease. All of these aspects should be incorporated in future models, ideally as spontaneously metastasizing mouse models that will be treated in a manner that closely relates to the clinical care—treating mice in mouse hospitals with neurosurgery, radiotherapy and different systemic therapies according to the mutational landscape of the tumor.

Another layer of complexion that recently has been acknowledged is the influence of host factors such as sex and age on various aspects of cancer and metastasis. Current in vivo models of experimental brain metastasis frequently neglect these aspects: Most studies in the field are conducted using only very young mice, not reflecting the clinical situation, in which brain metastasis occur rather in advanced stages of the disease, when cancer patients are usually older.^38^ In this context, experimentally it has been shown that the aging microenvironment can have a profound impact on the outgrowth of metastasis and therapy resistance.^87^ Similarly, recently it has been described how sex differences, particularly in the immune system, drive primary brain tumor growth and display distinct therapeutic vulnerabilities.^88^ Considering that the incidence of lung and melanoma brain metastasis is twice as high in men than it is in women,^38^ future models of brain metastasis should include both sexes and analyze the influence of perturbations separately to recognize these possible subtle differences.

### Novel Toolbox

Emerging technologies such as single-cell sequencing or spatial transcriptomics allow to decipher the previously unseen heterogeneity in both cancer cells and their surrounding microenvironment and have started to be applied to brain metastasis.^89^ While these approaches are aimed at elucidating the complexity of brain metastasis on a genetic level, novel imaging techniques such as tissue clearing have been developed that make it possible to follow metastatic cells in intact whole organs at single-cell level and subcellular resolution.^90^ In order to also study the progression of cancer cells in the brain in alive animals, intravital imaging through a skull window using 2-photon imaging allows longitudinal follow-up of single cells within the brain, for instance to track the fate of tumor initiating cells during early stages of brain metastasis formation.^91^

In addition to these descriptive approaches, new functional read-outs are necessary to characterize the local and systemic effects that brain metastasis have on their host. To this end, the development of new luciferases and their respective substrates, which do not overlap with the commonly in brain metastatic cell lines used firefly luciferase, enable the tracking of 2 distinct biological processes or cell populations by bioluminescence in live animals, for instance separately engrafted T-cells and tumor cells.^92^ Furthermore, as discussed earlier, the emerging notion of brain metastasis influencing neuro-cognitive functions and behavior is still far from being included in current models or considered in commonly used assays. Normally, experimental read-outs include different measurements of tumor growth, phenotyping of different cell populations and basic physiological parameters such as weight. Thus, there is a need to expand the horizon of experimental read-outs of preclinical brain metastasis models to behavioral and cognitive measurements. Here, longitudinal tracking of movement and behavior of animals over the course of disease using methods such as CAPTURE, which combines continuous motion capture with deep learning to infer the behavior of rodents,^93^ could lead to new insights of how brain metastasis influence normal brain function.

Lastly, thinking of new tools to enhance brain metastasis research should not only consider technical advancements but also novel model organisms. In addition to the earlier discussed rats which may represent a superior model for cancer neuroscience related questions, even larger animals could support mouse studies and function as an additional step between rodents and humans. Compared with humans or larger animals, the rodents brain outer cortex is smooth and lacks sulci, which improves drug delivery and therefore offers one explanation why many compounds which were successfully tested in small animals later fail in clinical trials.^94^ The immunocompromised strain of the Yucatan minipig has been previously used for engraftment of human glioblastoma xenografts; With the porcine brain gyrification as well as BBB physiology resembling much closer the human brain, it should have greater translational quality when it comes to preclinical testing of novel compounds.^94^

## Concluding Remarks

There is a wealth of human and murine models of brain metastasis, which are well characterized and constantly evolving, driven by a community-wide effort to improve this fatal disease. Novel tools and interdisciplinary collaboration with fields such as neuroscience, clinical research and genetics will help to generate a new generation of clinically relevant brain metastasis models, opening the door for the discovery of novel therapeutic targets.
